# A Single-Site Iron(III)-Salan Catalyst for Converting COS to Sulfur-Containing Polymers

**DOI:** 10.3390/polym9100515

**Published:** 2017-10-17

**Authors:** Ge-Ge Gu, Tian-Jun Yue, Zhao-Qian Wan, Rong Zhang, Xiao-Bing Lu, Wei-Min Ren

**Affiliations:** Key Laboratory of Fine Chemicals, Dalian University of Technology, 2 Linggong Road, Dalian 116012, China; gugege@mail.dlut.edu.cn (G.-G.G.); 201044103@mail.dlut.edu.cn (T.-J.Y.); wanzq@mail.dlut.edu.cn (Z.-Q.W.); rzhang@dlut.edu.cn (R.Z.); xblu@dlut.edu.cn (X.-B.L.)

**Keywords:** carbonyl sulfide, epoxide, iron catalyst, poly(monothiocarbonate)s

## Abstract

An iron(III) complex of tetradentate *N*,*N*′-disubstituted bis(aminophenoxide) (designated as salan, a saturated version of the corresponding salen ligand) with a sterically hindered organic base anchored on the ligand framework, can selectively mediate the conversion of carbonyl sulfide to sulfur-containing polymers by the copolymerization with epoxides. This single-site catalyst exhibits broad substrate scope, and the resultant copolymers have completely alternating structures. In addition, this catalyst is efficient in producing diblock copolymers, suggesting a living polymerization nature.

## 1. Introduction

Carbonyl sulfide (COS), as one of the largest components of organic sulfur compounds, is mainly produced from volcanic eruption and catabolism of biogenic volatile sulfur compounds, as well as human industrial activity [1]. COS is thought to be the most abundant sulfur-containing gas in the troposphere and therefore has been proved to be a major source of acid rain [2,3,4]. Apart from its effect on the environment, COS is normally regarded as a significant poison which can cause the deactivation of the industrial catalysts [5,6,7]. As a consequence, the removal of COS is one of the most important issues in the process of feed gas deep purification [8], while approaching the transformation of COS to desirable, economically competitive products remains a challenge [9,10].

Previously, Zhang and coworkers reported the transformation of COS to a degradable sulfur-containing polymer by means of the copolymerization with propylene oxide (PO) in the presence of a binary Cr(III)-salen complex/ionic ammonium salt catalyst system [11]. The formed copolymer has a high refractive index of 1.63. Subsequently, various poly(monothiocarbonate)s were prepared by the use of this Cr(III)-based catalyst systems or zinc–cobalt double metal cyanide complex [12,13,14,15,16,17]. We also developed a bifunctional Cr(III)-salen catalyst for COS/epoxides copolymerization in a remarkable activity, and no oxygen–sulfur exchange reaction was observed even at elevated temperatures [18]. This bifunctional catalyst was also efficient in converting COS to the semicrystalline polymer by the stereoregular copolymerization with enantiopure epichlorohydrin [19]. Recently, the semicrystalline poly(monothiocarbonate)s with good crystallization behavior were synthesized by the copolymerization of COS with achiral oxetane [20] or ethylene oxide [21] mediated by binary or bifunctional Cr(III)-based catalyst systems, respectively. More recently, colorless and highly transparent poly(monothiocarbonate)s were successfully obtained by the use of metal-free Lewis pair catalysts [21].

Iron, the most abundant transition metal in nature, is one of the most ideal metals for catalysis due to its low-cost and environmentally benign [22]. To date, various iron-catalyzed reactions for organic compounds and polymers have been realized [23]. For example, iron complexes have been reported as active catalysts for the coupling reaction of CO_2_ and epoxides [24,25,26,27,28]. In addition, the coordination of iron and sulfur exists widely in enzymes and synthetic ligands [29], indicating their good compatibility.

We have demonstrated that the Cr(III)-salen complexes bearing a sterically hindered organic base were excellent catalysts for COS/epoxides copolymerization to produce the corresponding poly(monothiocarbonate)s with high molecular weight and narrow monodispersity [18]. By contrast, the corresponding iron analogue was found to be inefficient in the same reactions. It is generally known that the ligand of salan (*N*,*N*′-disubstituted bis(aminophenoxide)) possess greater donor character than the corresponding salen ligand. Their chromium(III) and aluminum(III) complexes exhibited higher activity than the corresponding salen complexes in some cases [30,31]. On the other hand, we found the introduction of 1,5,7-triazabicyclo[4.4.0]dec-5-ene (designated as TBD) on the ligand framework could obviously decrease the catalyst loading and increase the polymer selectivity [18,32]. Motivated by these facts, herein we set out to develop an iron(III) complex of salan appending a TBD as single-site catalyst for efficiently converting COS to a wide range of sulfur-containing polymers by the copolymerization with epoxides.

## 2. Materials and Methods 

### 2.1. Synthesis of the Ligand

#### 2.1.1. Synthesis of Compound **5**

A solution of compound **4** (3.57 g) in dry tetrahydrofuran (THF) (40 mL) was stirred at room temperature and a solution of LiAlH_4_ (0.38 g) in dry THF (5 mL) was added dropwise. After stirring 3 h, water was added into the solution slowly. The resultant mixture was extracted by ethyl acetate three times. The combined organic phase was dried over anhydrous Na_2_SO_4_ and the solvent was removed under reduced pressure to give the desired product as a white solid. The compound **5** was used in the following reactions without further purification.

#### 2.1.2. Synthesis of Compound **6**

PBr_3_ (0.12 mL) was added to a stirred solution of compound **5** (3.41 g) in CHCl_3_ (50 mL). The white solid dissolved slowly after the reaction mixture was stirred at room temperature for 2 h. Cold water (30 mL) was added and the mixture was vigorously stirred for 2 min. The organic layer was separated and the aqueous layer was extracted with CHCl_3_ (2 × 50 mL). The combined organics were dried over anhydrous Na_2_SO_4_ and the solvent was removed under reduced pressure to give the desired product as a pale yellow solid. The compound **6** was used in the following reactions without further purification. Yield: 3.88 g (97%).

#### 2.1.3. Synthesis of the Ligand **3**

Compound **6** (1.53 g) in dry THF (100 mL) was added to a stirred solution of compound **7** (2.10 g) and then triethylamine (0.80 mL) was added dropwise. A white solid formed immediately, and stirring was continued for 2 h. The white solid was removed by filtration with water, and the solution was extracted by CH_2_Cl_2_ three times. The combined organics were dried over anhydrous Na_2_SO_4_. Removal of solvent under reduced pressure gave the crude product which was purified by chromatography (silica gel, dichloromethane/methanol = 20/1) to give the ligand **3** (2.10 g, 65% yield) as a white solid. ^1^H NMR (500 MHz, CDCl_3_) *δ* 7.20 (d, *J* = 2.0 Hz, 1H), 7.14 (s, 1H), 6.81 (t, *J* = 5.2 Hz, 2H), 3.70 (m, 6H), 3.53 (s, 2H), 3.37 (t, *J* = 5.8 Hz, 2H), 3.30 (dt, *J* = 12.4, 6.1 Hz, 4H), 2.69 (dd, *J* = 25.2, 17.2 Hz, 6H), 2.28 (s, 3H), 2.23 (s, 3H), 2.00 (dd, *J* = 18.4, 12.6 Hz, 6H), 1.38 (s, 9H), 1.27 (s, 9H), 1.26 (s, 8H). ^13^C NMR (125 MHz, CDCl_3_) *δ* 153.93, 152.85, 150.41, 141.78, 140.76, 135.52, 127.22, 123.55, 123.06, 121.03, 100.00, 62.65, 53.80, 50.58, 48.19, 47.47, 46.04, 41.63, 38.58, 34.80, 34.11, 33.93, 31.66, 31.63, 29.59, 27.39, 21.14, 20.78 (see Supporting Information, Figures S1 and S2). HRMS (*m/z*): Calcd. for [C_40_H_66_N_5_O_2_]^+^ ([3 + H]): 648.5217, found 648.5219.

### 2.2. Synthesis of Complexes

The ligand **3** (0.65 g, 1.0 mmol) and FeCl_3_ (0.17 g, 1.05 mmol) were dissolved in acetonitrile (10 mL), and the mixture was refluxed under nitrogen for 3 h. Then the reaction mixture was poured into diethyl ether (60 mL), and the organic layer was washed with aqueous saturated NH_4_Cl (3 × 60 mL) and brine (3 × 60 mL) followed by drying with anhydrous MgSO_4_. After filtration to remove solid impurities and drying agent, solvent was removed under vacuum, and thereby yielding a navy blue powder. The crude product was recrystallized from dichloromethane and hexane to give the complex **1**. Yield: 78%. HRMS (*m/z*): Calcd. for [C_40_H_64_N_5_O_2_FeCl]^+^ ([1 + H]): 737.4098, found 737.4098 (see Supporting Information, Figure S3).

Complex **1** (0.37 g, 0.5 mmol) was dissolved in CH_2_Cl_2_ (10 mL) in a 40 mL Schlenk vial wrapped in aluminum foil and then AgNO_3_ (0.090 g, 0.53 mmol) was added. The mixture was stirred for 24 h, and filtered to remove the Ag byproduct. Removal of solvent under reduced pressure gave the crude product which was further treated with the mixture of CH_2_Cl_2_ and hexane to give the complex **2** as a navy blue solid (0.36 g, 95%). HRMS (*m/z*): Calcd. for [C_40_H_64_N_6_O_5_Fe]^+^ ([2 + H]): 764.4288, found 764.4285 (see Supporting Information, Figures S4 and S5).

### 2.3. Representative Procedures for COS/Epoxides Copolymerization

A 50 mL autoclave equipped with a magnetic stirrer was heated to 120 °C under vacuum for 8 h, cooled under vacuum to room temperature and moved to a dry box. Complex **2** (38.2 mg, 0.05 mmol) and propylene oxide (5.8 g, 100 mmol, 2000 equivalent) were added in the autoclave. The autoclave was placed in a bath at 25 °C, and pressurized to COS (7.2 g, 120.0 mmol). After the allotted reaction time, the autoclave was cooled and the pressure was slowly vented. The reaction mixture was dried in vacuum at 50 °C for isolating the solvent and unreacted epoxide, then weighed to calculate the TOF (mol of product/mol of catalyst per hour) of the copolymerization. An aliquot was then taken from the resulting crude product for ^1^H NMR analysis (Varian, Palo Alto, CA, USA) to give the polymer selectivity. The crude polymer was suspended in a 10 mL CH_2_Cl_2_ and stirred for 10 min, then 50 mL methanol was added and filtered. This process was repeated 3–5 times to completely remove the catalyst. White precipitate was collected and dried in vacuum at 50 °C to constant weight. The obtained copolymer was analyzed by ^13^C NMR spectroscopy (Bruker, Billerica, MA, USA) and Gel Permeation Chromatography (GPC) (Agilent, Santa Clara, CA, USA).

## 3. Results and Discussion

**Figure 1:**
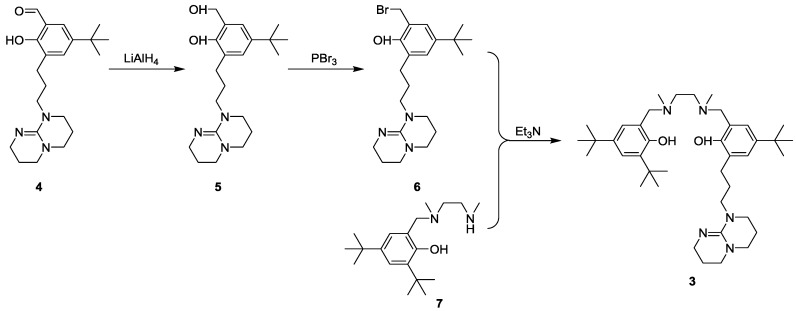
Synthetic routes for the ligand.

**Figure 2:**
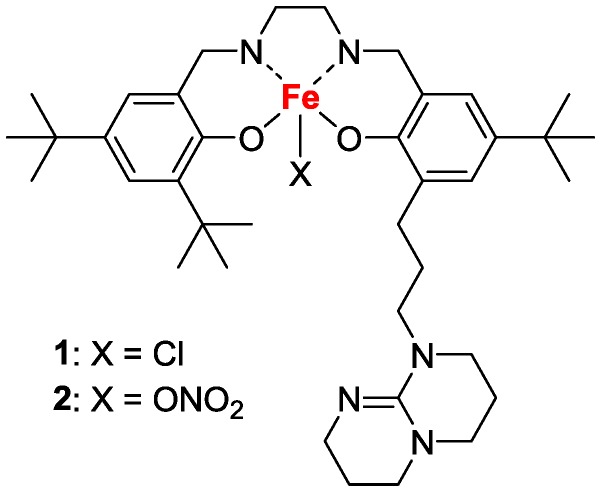
Structure of single-site iron(III) catalysts.

The salan ligand with an appended TBD was prepared by our previously reported method (Figure 1) [33]. The iron(III) complex **1** was synthesized by the reaction of the salan ligand and FeCl_3_ in acetonitrile. The further treatment with AgNO_3_ gave complex **2** (Figure 2). Since these iron complexes are easily dissolved in neat epoxides surveyed, the catalyzed copolymerization of COS and epoxides does not require any organic cosolvent. Only in some cases for complete conversion of epoxides, the addition of an organic solvent such as toluene is necessary for effective diffusion of the reactants.

**Figure 3:**
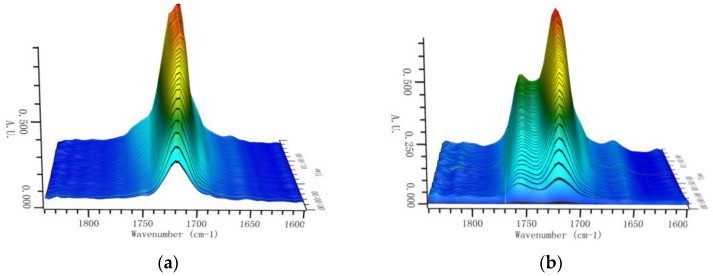
Three-dimensional stack plots of IR spectra recorded every 30 s during the copolymerization of PO with COS by the use of (**a**) complex **2** and (**b**) complex **1** as the catalyst at a [PO]/[catalyst] ratio of 2000, 80 °C, in the presence of 1,2-dimethoxyethane (DME) as solvent (PO/DME = 1/5, volume ratio).

Initially, PO was used as a model monomer to investigate the catalytic performance of the iron(III)-salan complexes. At a catalyst loading of 0.05 mol%, complex **1** exhibited a high activity of 1040 h^−1^ with a polymer selectivity of 94% at ambient temperature (Table 1, entry 1). The nucleophilicity of the axial anion of iron(III) complexes has a significant influence on COS/PO copolymerization. Although a change in the axial anion from Cl^−^ to less nucleophilic NO_3_^−^ decreases the TOF from 1040 to 700 h^−1^, an enhanced polymer selectivity of more than 99% was achieved at ambient temperature (entry 2). The resultant poly(propylene monothoicarbonate) has a completely alternating structure. Then, the bulk PO/CO_2_ copolymerizations were tested using complex **2** at increased temperatures and decreased catalyst loadings (entry 2–4). An activity of 5400 h^−1^ was achieved along with a polymer selectivity of 99% when the reaction was performed at 80 °C under a catalyst loading of 0.01 mol%. By way of contrast, complex **1** at the same conditions showed an increased activity for this coupling process with a TOF of 7290 h^−1^; however, the selectivity for copolymer formation was only 67% (entry 5). To better compare the discrepancy of the anion on the selectivity for polymer versus cyclic product formation, the copolymerization of PO and COS were conducted using complex **1** and **2** at 80 °C as monitored by in situ infrared spectroscopy (METTLER-TOLEDO, Columbus, OH, USA) (Figure 3). In order to prevent the intensity of (monothio)carbonate linkage resonance from growing too fast, the copolymerizations were performed in the presence of 1,2-dimethoxyethane as a solvent. Even the copolymerization mediated by complex **2** was performed over 2 h, only very weak *v*_C = O_ absorption at 1755 cm^−^^1^ assigned to the cyclic propylene monothiocarbonate was detected. In contrast, cyclic propylene monothiocarbonate was producted to a significant extent as the same conditions using the complex **1** as a catalyst. Notably, no oxygen−sulfur exchange reaction occurred in the present studies even at a high temperature of 80 °C, as confirmed by ^13^C NMR analysis. The single-site catalyst **2** was also proved to be efficient for the PO/COS copolymerization under a COS pressure of 0.1 MPa at 25 °C (entry 6). In the presence of an organic solvent such as toluene, quantitative conversion of the epoxide was achieved with >99% high polymer selectivity by a prolonged reaction time (entry 7). Furthermore, benzyl alcohol (BnOH) as a chain-transfer reagent was added into the reaction mixture for controlling the molecular weight of the copolymer. Under a PO/BnOH of 25/1 (molar ratio) and the quantitative conversion of the epoxide, the resultant poly(monothiocarbonate) has a molecular weight of 3000 g/mol (entry 8). The desired initiating group of BnO− was present in the polymer chain, determined by electrospray ionization mass spectrometry (see Supporting Information, Figure S6).

Having established that iron complex **2** is an effiecient catalyst for converting COS to poly(propylene monothiocarbonate), we then focused on its promising reactivity potential for synthesizing a variety of poly(monothiocarbonate)s by the copolymerization of COS and various epoxides. As expected, catalyst **2** was efficient in mediating the copolymerization of COS and monosubstituted terminal epoxides, including aliphatic epoxides, aryl epoxide, halogenated terminal epoxide and ether-containing terminal epoxides, to selectively afford the corresponding sulfur-containing polymers with more than 99% polymer selectivity at 25 °C (Table 2, entries 1–8). Moreover, this catalyst can selectively convert COS to poly(alicyclic monothiocarbonate)s from alicyclic epoxides at 50 °C (entries 9–12). All the resultant copolymers have completely alternating structure, and no oxygen-sulfur exchange reaction occurred with regard to all terminal and alicyclic epoxides (see Supporting Information, Figures S7–S14).

**Scheme 1:**
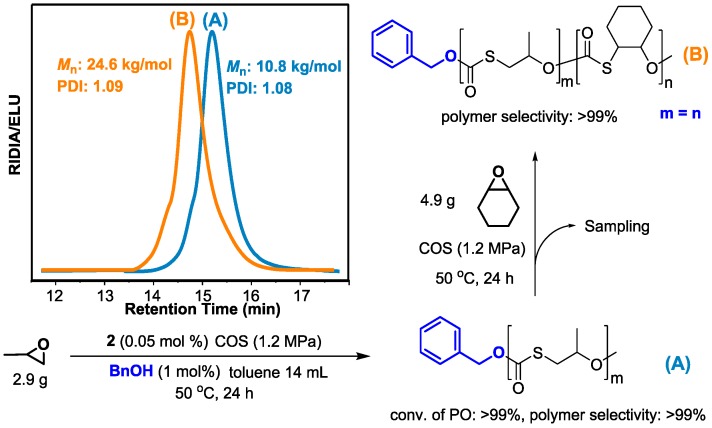
The synthesis and characterization of diblock copolymers of poly(propylene monothiocarbonate) and poly(cyclohexylene monothiocarbonate).

Because of the living polymerization nature, we further investigated catalyst **2** for the synthesis of diblock copolymer of poly(propylene monothiocarbonate) and poly(cyclohexylene monothiocarbonate). In order to obtain the block copolymer as pure as possible, benzyl alcohol as chain-transfer reagent was employed to minimize the effect of trace water from the reactants. The block copolymer was produced by stepwise addition of the two different epoxides, PO and cyclohexene oxide (CHO) under 50 °C (Scheme 1). Quantitative conversion of epoxides was achieved with more than 99% polymer selectivity under a toluene/epoxides ratio of 2/1. The diblock copolymer formation was detected by the shift of the GPC trace relative to the PO/COS copolymer trace (Scheme 1). Based on ^1^H NMR analysis, the resulting diblock copolymer shows more than 99% monothiocarbonate linkages (see Supporting Information, Figure S15). No oxygen–sulfur exchange reaction occurred as confirmed by ^13^C NMR spectroscopy (see Supporting Information, Figure S16).

**Figure 4:**
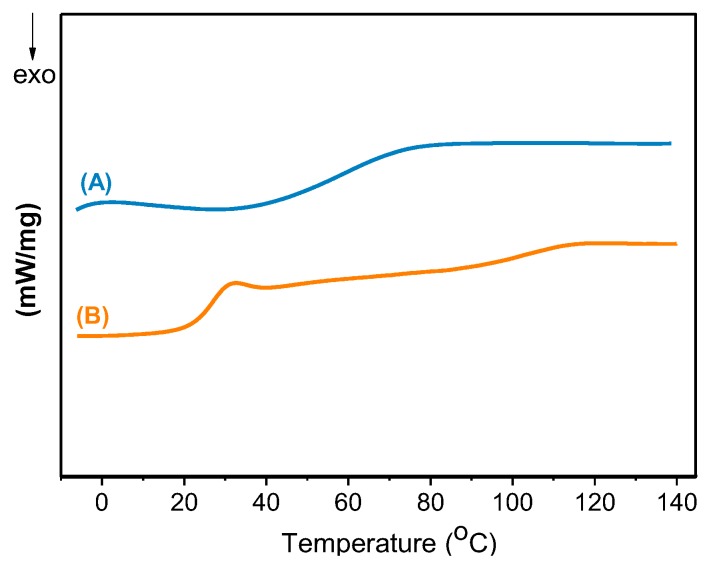
DSC thermograms of (**A**) the diblcok polymer containing 50 mol% PO/COS copolymer segment, (**B**) the blend of PO/COS copolymer and CHO/COS copolymer (1/1 molar ratio in monothiocarbonate unit).

The thermal behavior of the diblock copolymer was studied by using Differential Scanning Calorimeter (DSC) (NETZSCH, Bavaria, Germany) in a nitrogen flow. Similar with the reported PO/CHO/COS terpolymer [15], only one glass transition temperature (*T*_g_) of 68.5 °C was found in the diblock copolymer (Figure 4A). But it is worth noting that the baseline shift is significantly broader than that of the COS/PO/CHO terpolymer. By way of contrast, the thermogram of the blend composed of PO/COS copolymer and CHO/COS copolymer shows two baseline shifts (Figure 4B), one *T*_g_ at 25.6 °C (attributable to the PO/COS copolymer) and the other *T*_g_ at 115.1 °C (attributable to the PO/COS copolymer). This result suggests that PO/COS copolymer and CHO/COS copolymer segments in the diblock polymer have good miscibility in comparison with the blend.

## 4. Conclusions

We have demonstrated that the salan complex of cheap, earth-abundant, and nontoxic iron(III) metal was an efficient living polymerization catalyst for converting COS to sulfur-containing polymers by the copolymerization with epoxides. The single-site catalyst displays a wide substrate scope and functionality tolerance, including aliphatic, aryl, halogenated, ether-containing terminal epoxides as well as alicyclic epoxides. Furthermore, complete consumption of the epoxide with high copolymer selectivity was achieved in the presence of an organic solvent, allowing for the synthesis of the diblock copolymer of poly(propylene monothiocarbonate) and poly(cyclohexylene monothiocarbonate).

## Figures and Tables

**Table 1:**
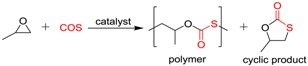
Iron(III)-salan complexes mediated COS/PO copolymerization *^a^*.

Entry	Catalyst	[PO]/[cat]	Temperature (°C)	Time (h)	TOF *^b^* (h^−1^)	Selectivity *^c^* (% Polymer)	*M*_n_ *^d^* (kg/mol)	PDI *^d^*
1	1	2000	25	1.0	1040	94	33.3	1.15
2	2	2000	25	1.0	700	>99	22.3	1.14
3	2	5000	50	0.5	2820	>99	33.6	1.17
4	2	10,000	80	0.5	5400	99	56.9	1.23
5	1	10,000	80	0.5	7290	67	48.9	1.28
6 *^e^*	2	2000	25	10.0	136	>99	57.4	1.17
7	2	2000	50	24.0	83	>99	65.6	1.18
8 *^f^*	2	2000	50	24.0	83	>99	3.0	1.09

*^a^* Copolymerizations were conducted in a preheated autoclave at a COS/PO of 1.2/1 (molar ratio) unless noted otherwise. Reactions were performed in neat PO for entries 1–6 and in the presence of toluene as solution with a toluene/PO of 2:1 (*v*/*v*) for entries 7 and 8. The monothiocarbonate linkages of the resulted copolymers are all >99% based on ^1^H NMR spectroscopy. No oxygen–sulfur exchange reaction occurred as confirmed by ^13^C NMR spectroscopy. *^b^* TOF = mol of product/mol of catalyst per hour. *^c^* Determined using ^1^H NMR spectroscopy. *^d^* PDI = *M*_w_/*M*_n_. Determined by gel permeation chromatography in THF, calibrated with polystyrene standards. *^e^* The copolymerization was carried out at 0.1 MPa COS. *^f^* Benzyl alcohol (BnOH) was added with a PO/BnOH of 25/1 (molar ratio). The theoretical molecular weight was calculated as: *M*_n_,theor = [TOF]·[time]·118.02·[1 + r]−^1^ (r = *n*_BnOH_/*n*_cat_).

**Table 2:**
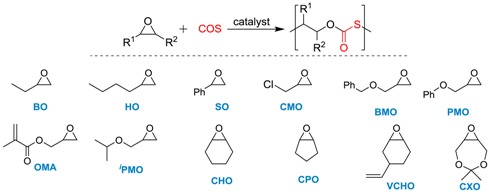
COS/Various epoxides copolymerization results *^a^*.

Entry	Epoxide	Time (h)	TOF *^b^* (h^−1^)	*M*_n_ *^c^* (kg/mol)	PDI *^c^*
1	BO	3.0	343	43.6	1.12
2	HO	5.0	216	49.0	1.15
3	SO	24.0	39	14.6	1.18
4	CMO	24.0	75	5.3	1.53
5	OMA	2.0	490	25.8	1.19
6	iPMO	3.0	368	35.3	1.25
7	BMO	3.0	208	24.6	1.12
8	PMO	3.0	268	31.2	1.18
9	CHO	3.0	442	50.5	1.10
10	CPO	24.0	23	20.0	1.21
11	VCHO	3.0	267	35.8	1.11
12	CXO	24.0	10	12.5	1.21

*^a^* Copolymerizations were mediated by complex **2** in neat epoxides at a COS/epoxide/**2** of 2400/2000/1 (molar ratio) under 25 °C for entries 1−8 and 50 °C for entries 9−12. No cyclic monothiocarbonates and ether linkages were found by ^1^H NMR spectroscopy. No oxygen–sulfur exchange reaction occurred as confirmed by ^13^C NMR spectroscopy. *^b^* TOF = moles of product per mole of catalyst per hour. *^c^* PDI = *M*_w_/*M*_n_. Determined by gel permeation chromatography in THF, calibrated with polystyrene standards.

## Supplementary Materials

The following are available online at www.mdpi.com/2073-4360/9/10/515/s1, characterization of the complexes and poly(monothicarbonte)s. Figure S1: ^1^H NMR spectrum of the ligand; Figure S2: ^13^C-NMR spectrum of the ligand; Figure S3: The HRMS spectrum of complex **1**; Figure S4: The HRMS spectrum of complex **2**; Figure S5: UV-Vis absorption spectra of a dichloromethane solution of complex 2 (*c* = 6.2 × 10^−6^ M); Figure S6: Electrospray ionization mass spectrum of poly(propylene monothiocarbonate) with the initiating group of BnO−; Figure S7: ^1^H NMR spectrum of OMA/COS copolymer; Figure S8: ^13^C NMR spectrum of OMA/COS copolymer; Figure S9: 1H NMR spectrum of ^i^PMO/COS copolymer; Figure S10: ^13^C NMR spectrum of ^i^PMO/COS copolymer; Figure S11: ^1^H NMR spectrum of VCHO/COS copolymer; Figure S12: ^13^C NMR spectrum of VCHO/COS copolymer; Figure S13: ^1^H NMR spectrum of CXO/COS copolymer; Figure S14: ^13^C NMR spectrum of CXO/COS copolymer; Figure S15: ^1^H NMR spectrum of diblock copolymers of poly(propylene monothiocarbonate) and poly(cyclohexylene monothiocarbonate); Figure S16: ^13^C NMR spectrum of diblock copolymers of poly(propylene monothiocarbonate) and poly(cyclohexylene monothiocarbonate).
