# Supplementary material for: A Single-Site Iron(III)-Salan Catalyst for Converting COS to Sulfur-Containing Polymers
Source: Polymers (Basel). 2017 Oct 17;9(10):515. doi: 10.3390/polym9100515 (PMC6418724; doi:10.3390/polym9100515)
Supplement: Supplementary file 1 [file polymers-09-00515-s001.pdf]

Supplementary Materials: A Single-site Iron(III)-salan Catalyst for Converting COS to Sulfur-containing Polymers

Ge-Ge Gu, Tian-Jun Yue, Zhao-Qian Wan, Rong Zhang, Xiao-Bing Lu and Wei-Min Ren*

General information

All manipulations involving air- and/or water-sensitive compounds were carried out in glove box or under dry nitrogen using standard Schlenk techniques. The epoxides were distilled under a nitrogen atmosphere from CaH_2 prior to use. Carbonyl sulfide (COS) (99.995%) was purchased from Dalian Institute of Special Gases.

^{1}H NMR and ^{13}C NMR spectra were recorded on a Varian INOVA-500 MHz type (1H, 500 MHz; ^{13}C, 125 MHz) spectrometer. Their peak frequencies were referenced versus an internal standard (TMS) shifts at 0 ppm for both ^{1}H NMR and ^{13}C NMR. While the solvent, $CDCl_3$ was recorded at 7.26 and 77.4 ppm for ^{1}H NMR and ^{13}C NMR, respectively.

Gel Permeation Chromatography Molecular weights and molecular weight distributions of copolymers were measured by gel permeation chromatography (GPC) analysis at 35 °C and a flow rate of 1.0 mL/min, with THF as the eluent, on a Agilent 1260 instrument coupled with an Agilent RI detector and equipped with four PL gel 5 m mixed-C columns. The sample concentration was about 0.1%, and the injection volume was 50 μL. The curve was calibrated using monodisperse polystyrene standards covering the molecular weight rage from 580 to 460000 Da.

Differential Scanning Calorimetry (DSC) The analysis of DSC was carried out with a NETZSCH DSC 206 thermal analyzer.

Electrospray Ionization Mass Spectrometry (ESI-MS) ESI mass spectrum of the copolymer in positive ion mode were carried out using a ESI Q-TOF (Waters, Milford, Massachusetts, USA) mass spectrometer equipped with an orthogonal electrospray source (Z-spray) and referenced against the sample of $(m/z)^+ = 556.2771$ (Sample cone = 5 V).

In situ infrared spectroscopy in a typical experiment, a 100 mL stainless steel Parr autoclave reactor, modified with a ZnSeW AR window to allow for the use of an ASI ReactIR 45 system equipped with a MCT detector and 30 bounce DiCOMP in situ probe, is heated to the desired temperature. In this manner, a single 256-scan background spectrum was collected. The catalyst dissolved in epoxide of 20 mL was then injected into the reactor via the injection port. The reactor was pressurized to COS as the IR probe began collecting scans. The infrared spectrometer was set up to collect one spectrum every 30 seconds over a certain period. Profiles of the absorbance at approximately 1715 cm^{-1} υ(C=O) corresponding to poly(monothiocarbonate) and around 1755 cm^{-1} υ(C=O) corresponding to cyclic monothiocarbonate with time were recorded and used to provide initial reaction data for analysis.

1. The Characterization of the Ligand

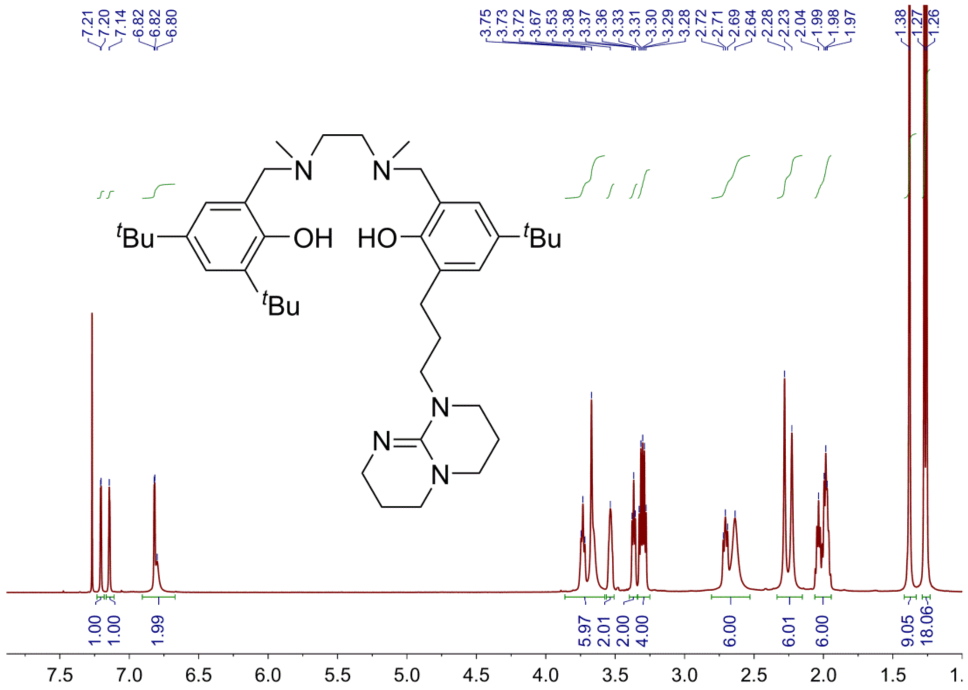

Figure S1. ^{1}H NMR spectrum of the ligand.

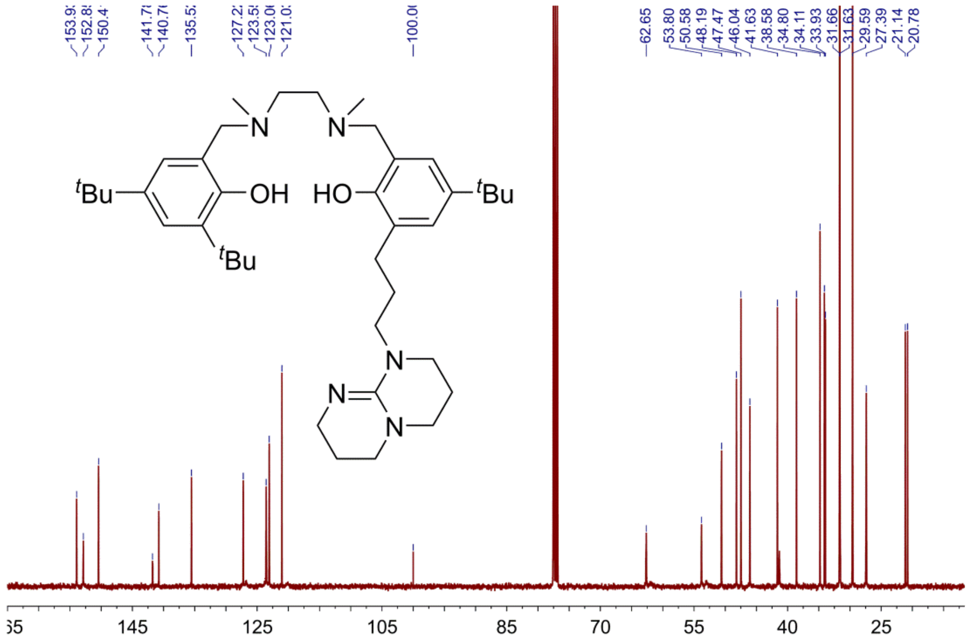

Figure S2. ^{13}C NMR spectrum of the ligand.

2. The characterization of the complexes

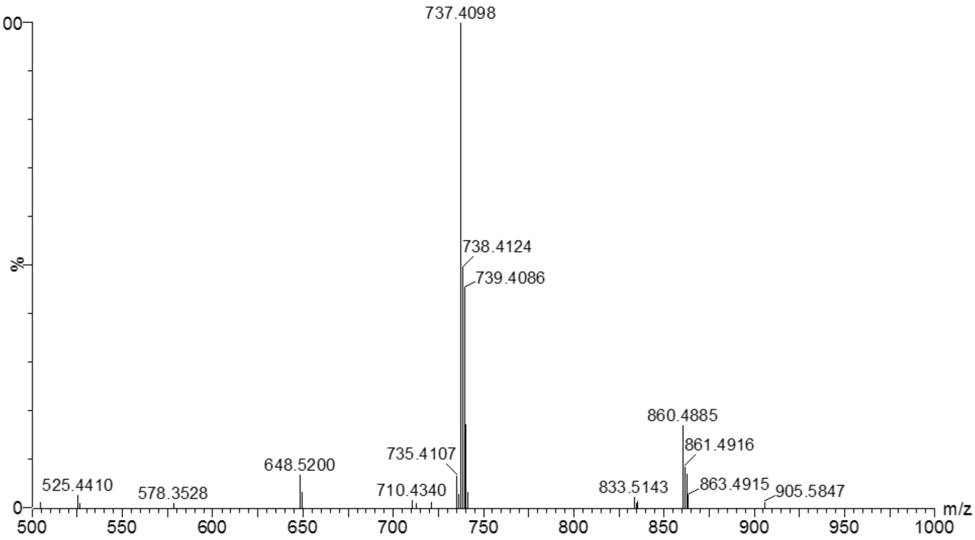

Figure S3. The HRMS spectrum of complex **1**.

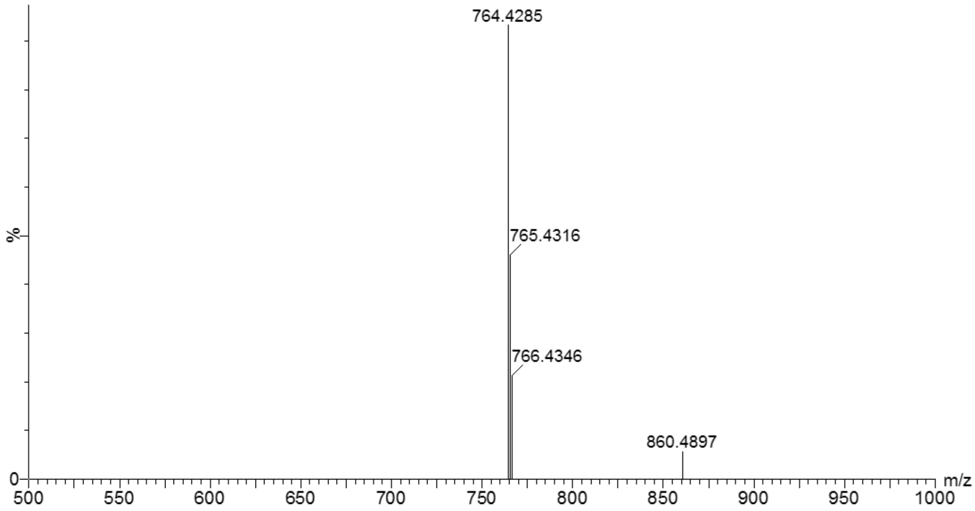

Figure S4. The HRMS spectrum of complex **2**.

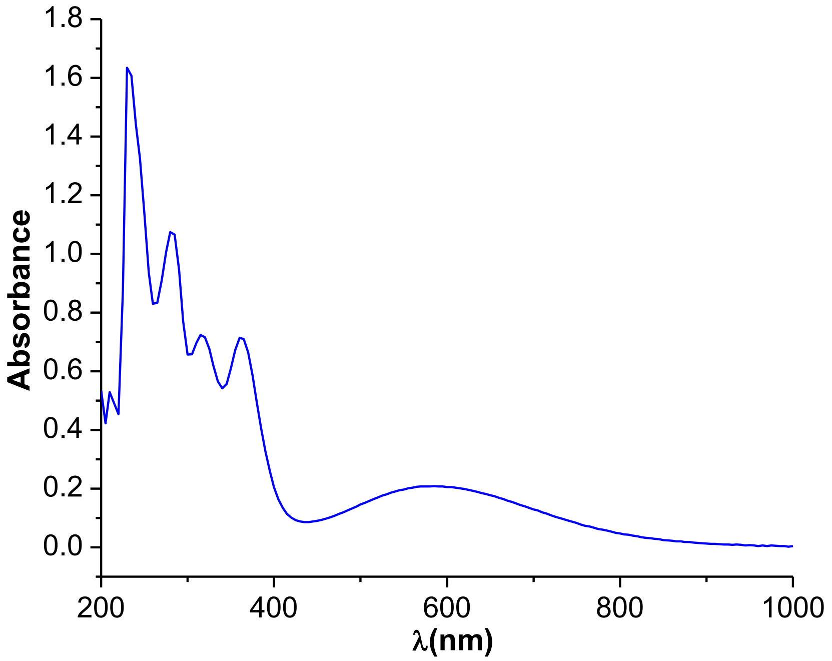

Figure S5. UV-Vis absorption spectra of a dichloromethane solution of complex **2** (*c* = 6.2 × 10^{-6} M). The UV/Vis spectrum of 2 in dichloromethane solution exhibits absorbed band at 600 nm.

3. The Characterization of Poly(propylene monothiocarbonate) Obtained from Catalyst 2-Mediated PO/COS Copolymerization under a [PO]/[BnOH] Ratio of 25/1

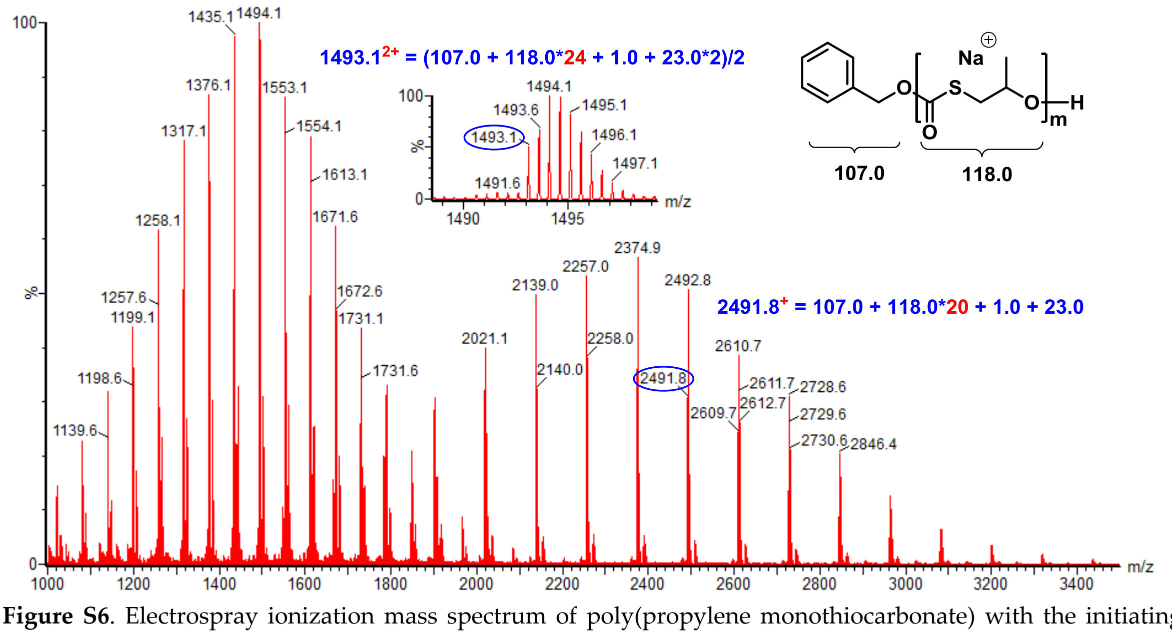

Figure S6. Electrospray ionization mass spectrum of poly(propylene monothiocarbonate) with the initiating group of BnO−, which was obtained from catalyst **2**-mediated PO/COS copolymerization under a [PO]/[BnOH] ratio of 25/1 at 50 °C.

4. The Characterization of the Resultant Poly(monothiocarbonate)s from the Copolymerization of COS and Various Epoxides

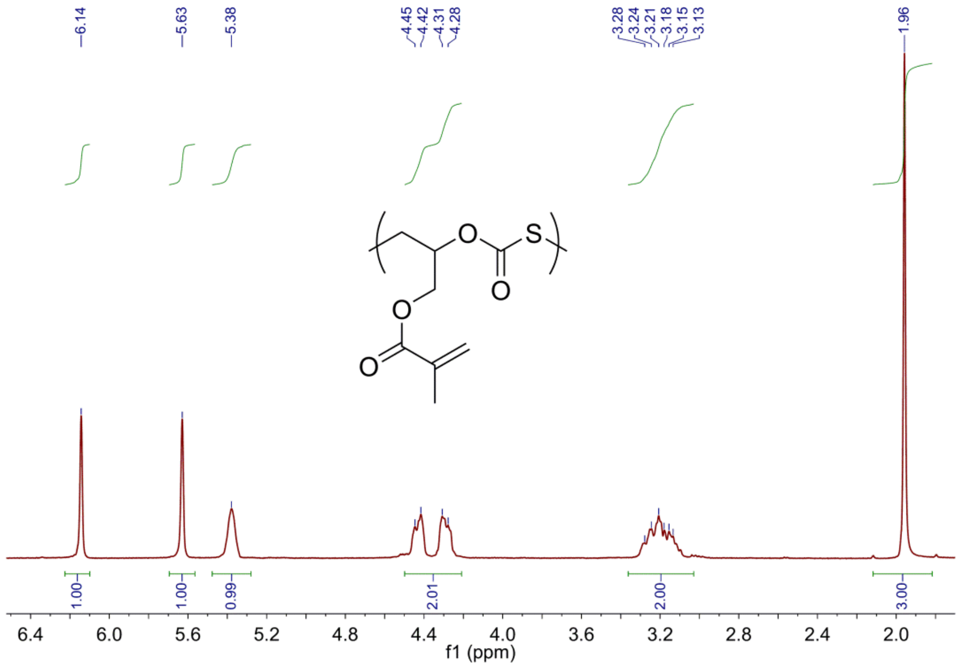

Figure S7. ^{1}H NMR spectrum of OMA/COS copolymer.

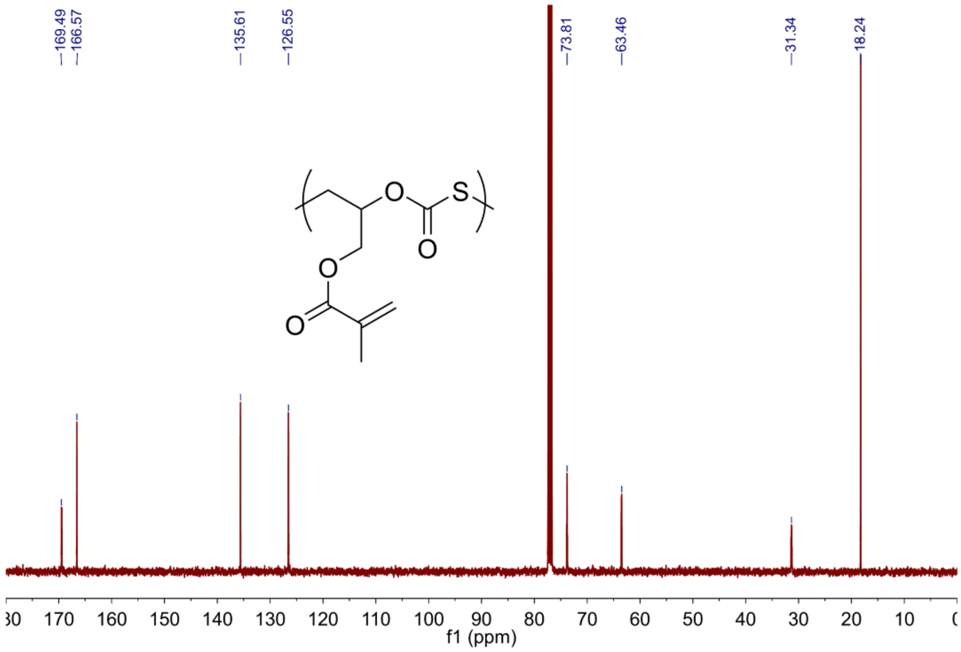

Figure S8. ^{13}C NMR spectrum of OMA/COS copolymer.

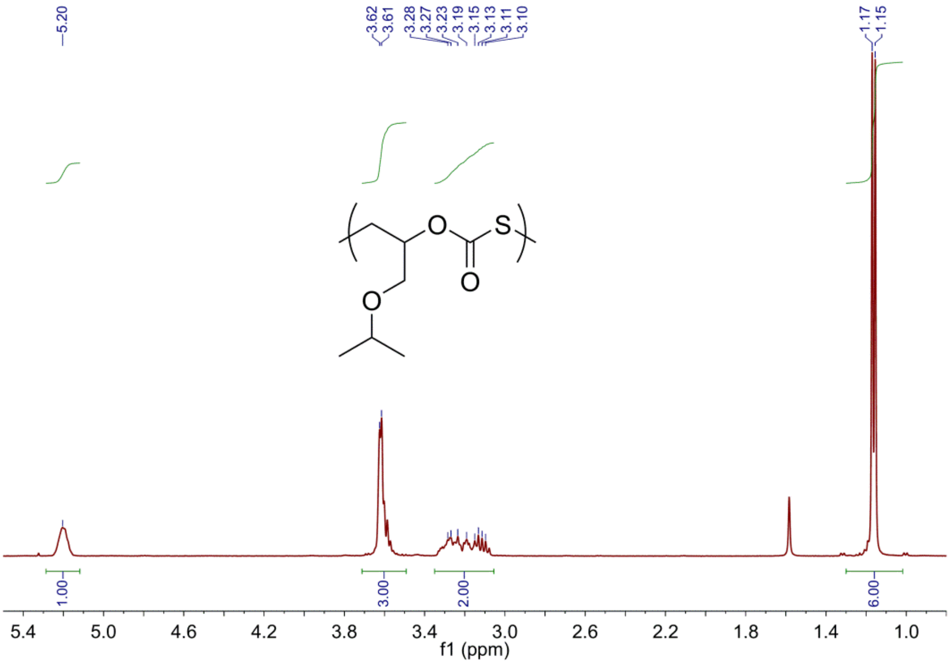

Figure S9. ^{1}H NMR spectrum of ^{i}PMO/COS copolymer.

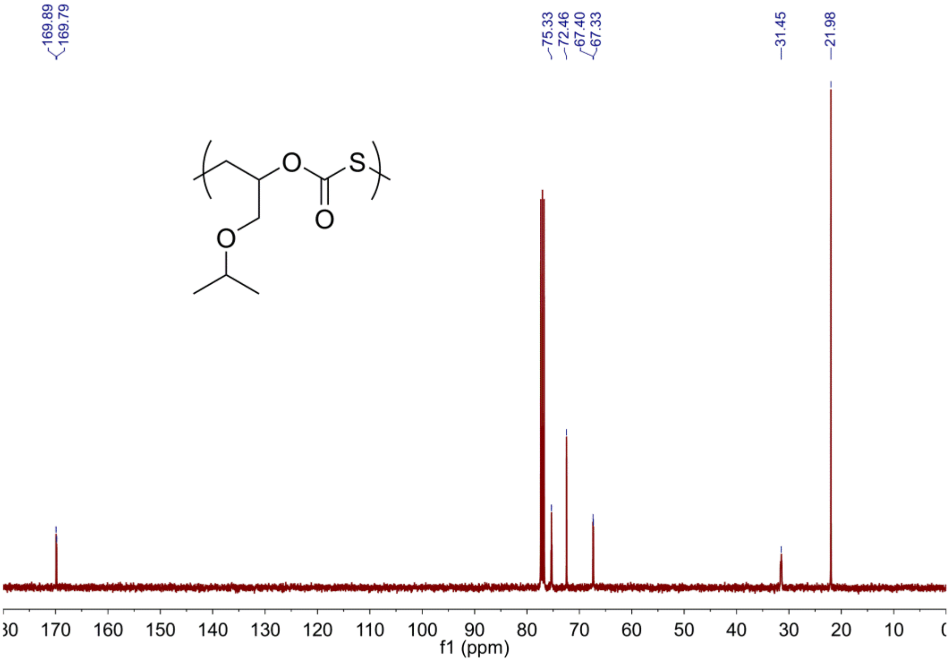

Figure S10. ^{13}C NMR spectrum of ^{i}PMO/COS copolymer.

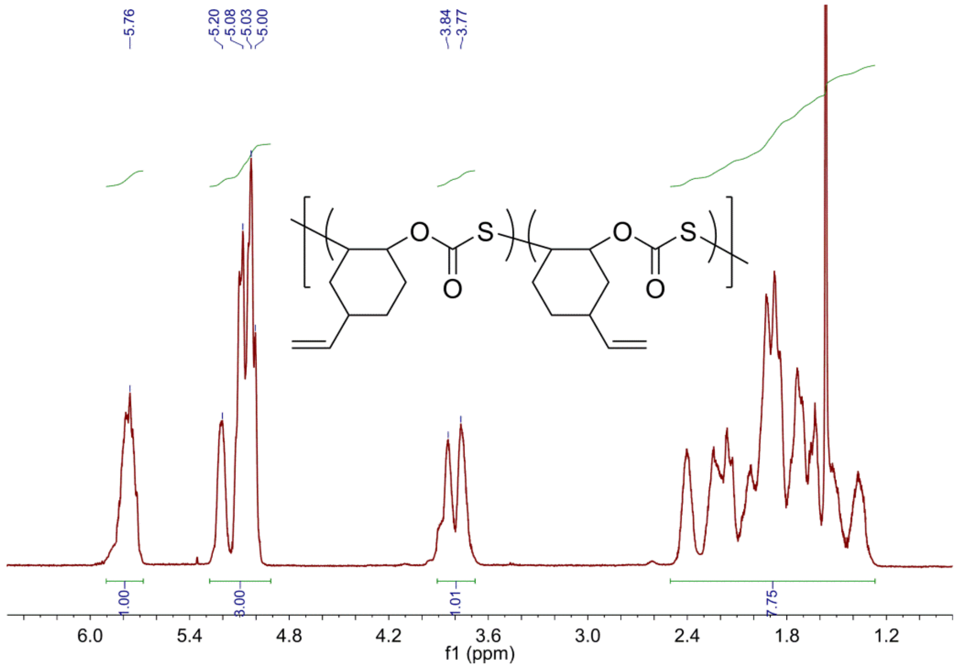

Figure S11. ^{1}H NMR spectrum of VCHO/COS copolymer.

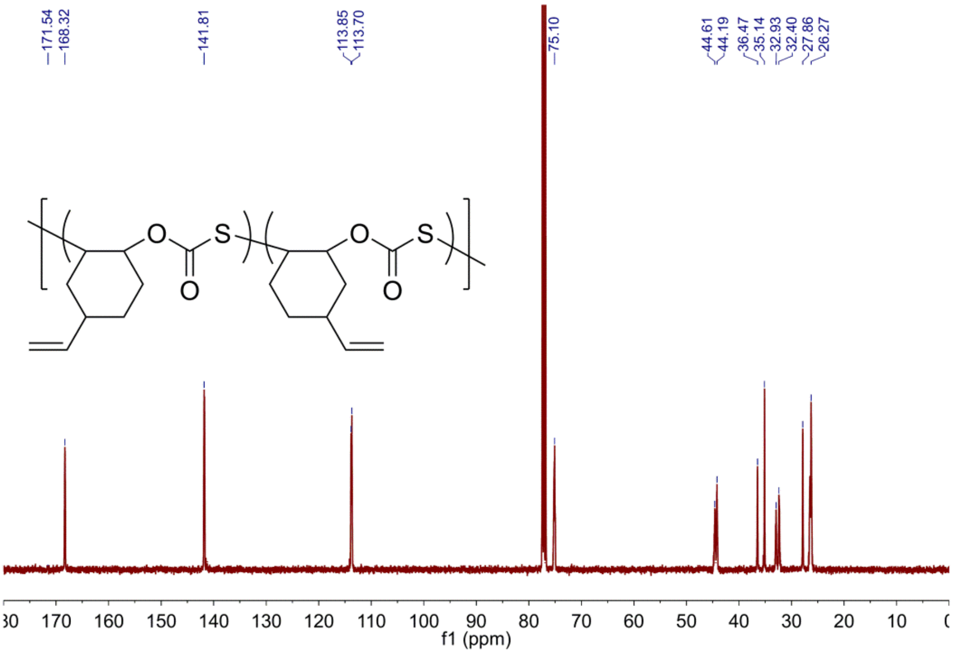

Figure S12. ^{13}C NMR spectrum of VCHO/COS copolymer.

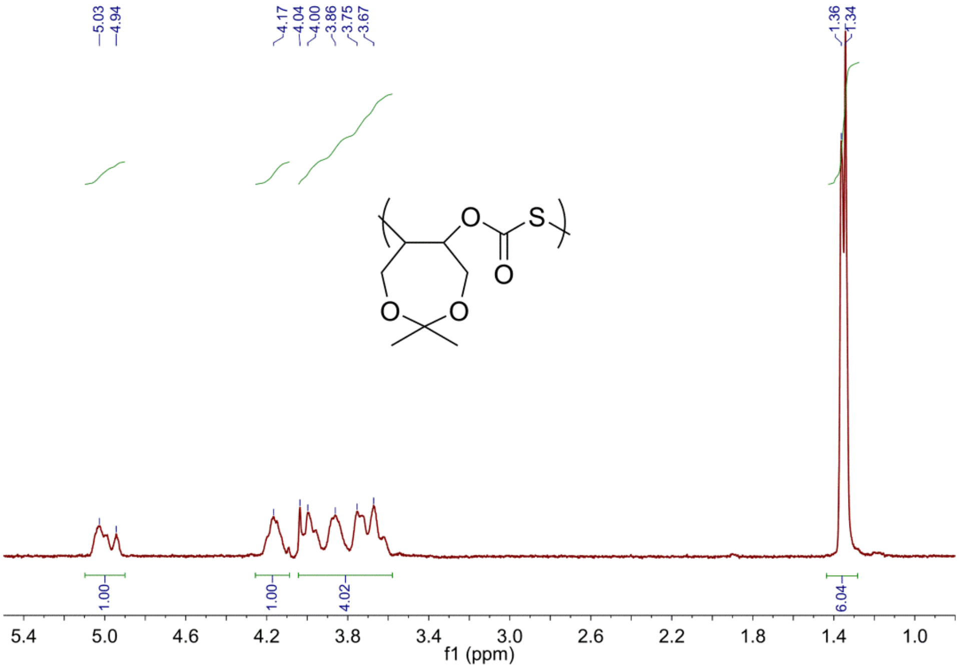

Figure S13. ^{1}H NMR spectrum of CXO/COS copolymer.

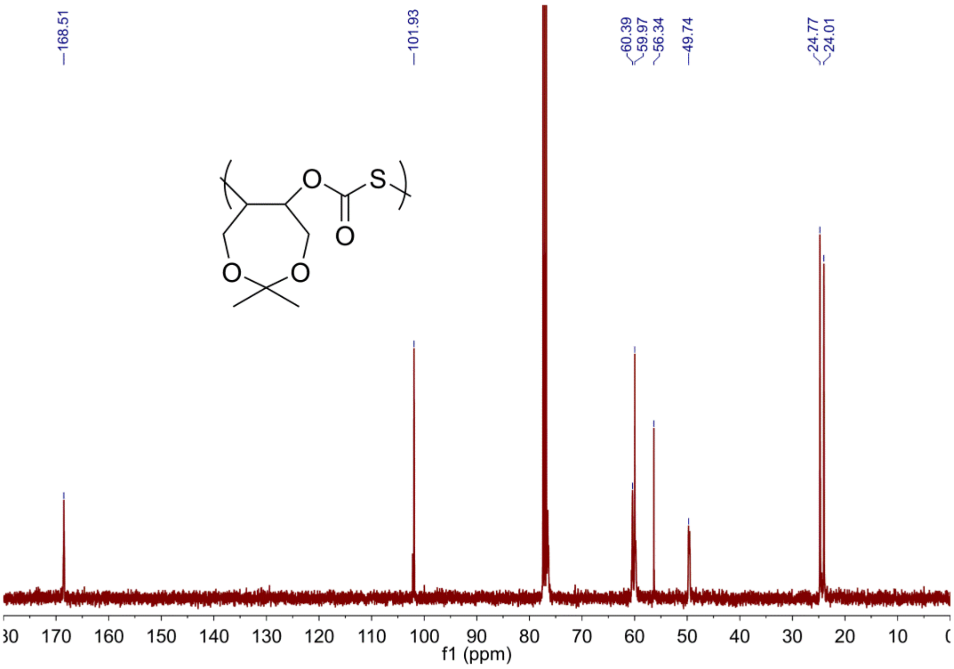

Figure S14. ^{13}C NMR spectrum of CXO/COS copolymer.

5. The Procedures for the Formation of Diblock Copolymers

To a stirred mixture of complex **2** (0.025 mmol, 1 equiv.) was dissolved in propylene oxide (50 mmol, 2000 equiv.) to form a red-brown solution in a nitrogen atmosphere. The mixture solution was added benzyl alcohol (0.5 mmol) and toluene (14 mL), then charged into a pre-dried 100 ml autoclave equipped with a magnetic stirrer under a COS atmosphere. After COS (1.2 MPa) was introduced, the reaction mixture was stirred at 50 °C for 24 h. Then a small amount of the polymerization mixture was picked out to determine the conversion of propylene oxide (>99%), the polymer selectivity (>99%), molecular weight (M_n = 10.8 kg·mol^{-1}), and molecular weight distribution (M_w/M_n = 1.08). The rest of the polymerization mixture was added cyclohexene oxide (50 mmol, 2000 equivalent.). The

reaction mixture was stirred at 50 °C for 24 h. The COS pressure was released, and then a small amount of the polymerization mixture was picked out to determine the polymer selectivity (>99%), molecular weight (M_n = 24.6 kg·mol^{-1}), and molecular weight distribution (M_w/M_n = 1.09). The crude polymer was suspended in 50 mL CH_2Cl_2 and stirred for 10 min at 50 °C. When the mixture was cooled to room temperature, 200 mL methanol was added and filtered. This process was repeated 3-5 times to completely remove the catalyst. The white precipitate was collected and dried in vacuum at 50 °C to constant weight. The obtained copolymer was analyzed by ^{1}H NMR and ^{13}C NMR spectroscopy, as well as DSC.

6. The Characterization of Diblock Copolymers

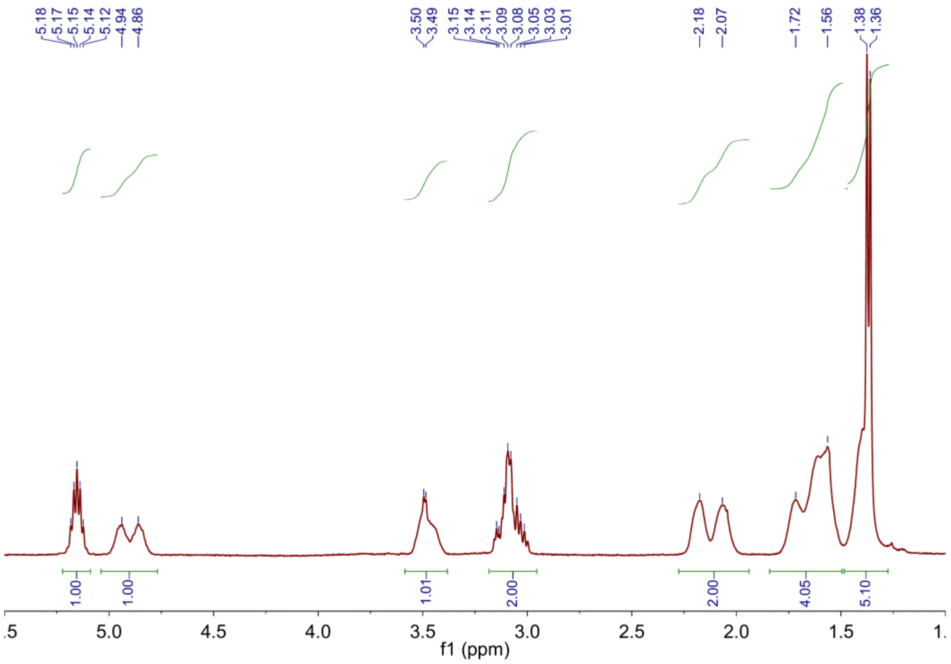

Figure S15. ^{1}H NMR spectrum of diblock copolymers of poly(propylene monothiocarbonate) and poly(cyclohexylene monothiocarbonate).

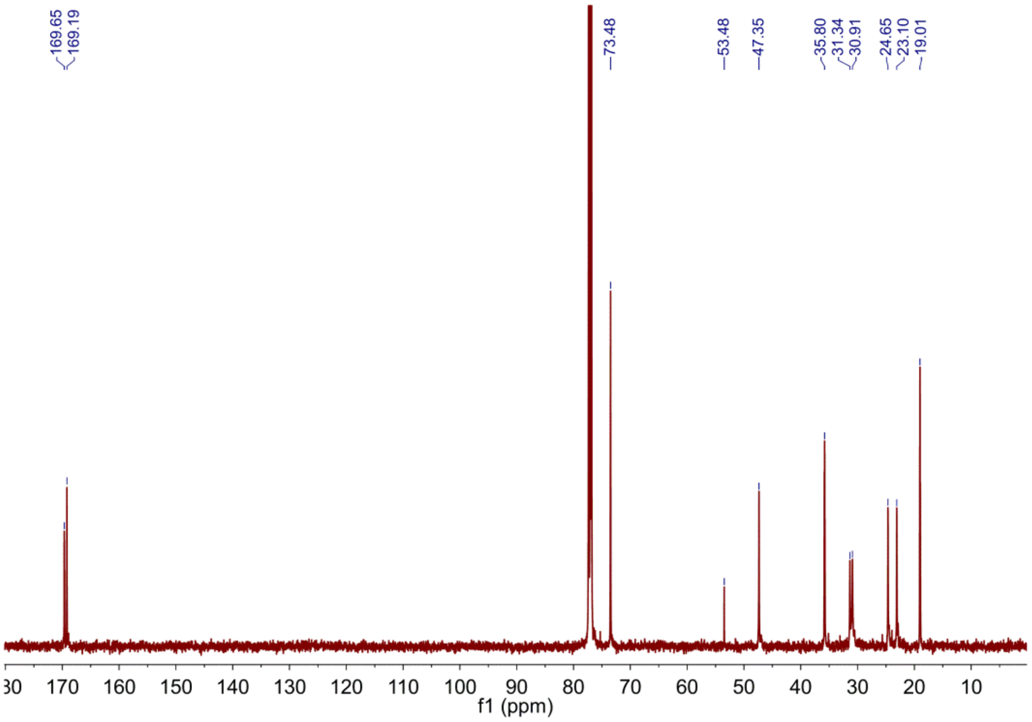

Figure S16. ^{13}C NMR spectrum of diblock copolymers of poly(propylene monothiocarbonate) and poly(cyclohexylene monothiocarbonate).
